# Case Report of Bilateral 3-4 Metatarsal Syndactyly in a Pet Rabbit

**DOI:** 10.1155/2016/6957101

**Published:** 2016-06-19

**Authors:** M. Gallego, L. Avedillo

**Affiliations:** ^1^Centro Veterinario Madrid Exóticos, Calle Meléndez Valdés 17, 28015 Madrid, Spain; ^2^Centro Veterinario Salud Animal, Calle de la Iglesia 10, Griñón, 28971 Madrid, Spain

## Abstract

We report the first case of spontaneous syndactyly reported in a pet rabbit. Syndactyly only caused an atypical gait in the rabbit. The radiological study revealed bilateral 3rd and 4th metatarsal bones fused in its entire length preserving normal joint surfaces resembling syndactyly type Ia. The cause of this congenital malformation was unknown.

## 1. Introduction

Syndactyly is a congenital malformation in which two or more fingers are joined because they fail to separate or fuse during limb development. The fusion of carpal, tarsal, metacarpal, and metatarsal bones is included in the term syndactyly, as they often occur together [1]. In human and veterinary medicine syndactyly has been classified into several types: simple, which affects only soft tissue; complex, involving synostosis; complete, involving entire length synostosis of the fused bones; incomplete, when synostosis does not comprise the entire bone length; complicated, when syndactyly appears with other malformations in the same individual; and uncomplicated if there are no more malformations [1–3].

Congenital syndactyly has been observed in dogs, cats, sheep, pigs, and cows [3–8]. Although in dogs and cats are cited isolated cases in the literature, it has been considered by some authors that syndactyly is hereditary in these species [2, 3]. Syndactyly in dogs can be complicated: in a family of Australian Shepherd dogs a multiple inherited teratologic syndrome that was lethal to males has been reported [7]. Although in sheep heritability has been suggested, it is in cows in the species in which hereditary syndactyly, in autosomal recessive form, is best described [4, 6, 8].

In humans syndactyly is associated with a mutation in the HoxD-13 gene in a syndrome called synpolydactyly, where bone fusions and duplications in hands and feet occur [9]. There are other causes of syndactyly in people; in fact, in more than 90 multiple malformation syndromes syndactyly is present [1, 10].

Mice genetically modified for experimental embryology have been employed to investigate the embryonic development of the limbs. In these studies syndactyly appeared in the offspring occasionally. Syndactyly was found both in mice lacking laminin alpha-5 chain gene and in mice null for fibrillin-2 gene. Retinoic acid receptor gene mutation in mouse also caused syndactyly. Inhibition of interdigital cell death was observed in functional cell proteins TGF Beta-2/TGF Beta-3 double knockout mice. Mice null for both Apaf1 and bax/bak, respectively, gene, and cell proteins implicated in apoptosis developed soft tissue syndactyly [11, 12].

In reference texts of pet rabbit medicine the following congenital conditions are cited: cryptorchidism, incisor malocclusion, spinal deformities, incomplete tracheal rings, splay leg, uterine malformations, renal agenesis, congenital cardiac disease, polycystic kidney disease, hereditary ataxia, glaucoma, cataracts, lymphoma, and cutaneous asthenia [13–15]. However there are few congenital diseases described in pet rabbits; in the other conditions literature refers to laboratory rabbits or to observations in daily clinical practice. Except for the congenital incisor malocclusion, which is best described [16], there are only sparse case reports: two reports of congenital ventricular septal defects, one case of incomplete tracheal rings, various cases of cutaneous asthenia, one case of bilateral tibial agenesis, two cases of congenital uterine malformations, congenital cataracts, and one corneal dermoid [17–26]. Syndactyly has not been reported in pet rabbits.

Previously, syndactyly has only been observed in laboratory rabbits on studies to assess the teratogenic effects in fetuses of different compounds administered to pregnant does. Vitamin A, 6-aminonicotinamide, hydroxyurea, thalidomide, and cyclophosphamide resulted in offspring syndactyly, in addition to other malformations [27–30]. Syndactyly was induced in rabbits as a result of aberrant scarring after causing traumatic injury on fetuses in utero or caused by uterine puncture to obtain amniotic fluid [31, 32]. After an exhaustive literature review, the authors founded only a citation of spontaneous syndactyly in a laboratory rabbit, a fetus of New Zealand white rabbit that was part of one of the 33 malformated individuals from a total of 2821 control rabbits from a breeding laboratory colony [33].

## 2. Case Histories

A three-year-old mixed breed pet male rabbit weighing 1.6 kg was admitted in a veterinary surgeon for routine sterilization. During the consultation, the owner referred an unusual walking since he bought it in a store when it was 6 weeks old. A detailed physical examination revealed no abnormalities. A radiological study of the hindlimbs was accepted by the owner.

**Figure 1:**
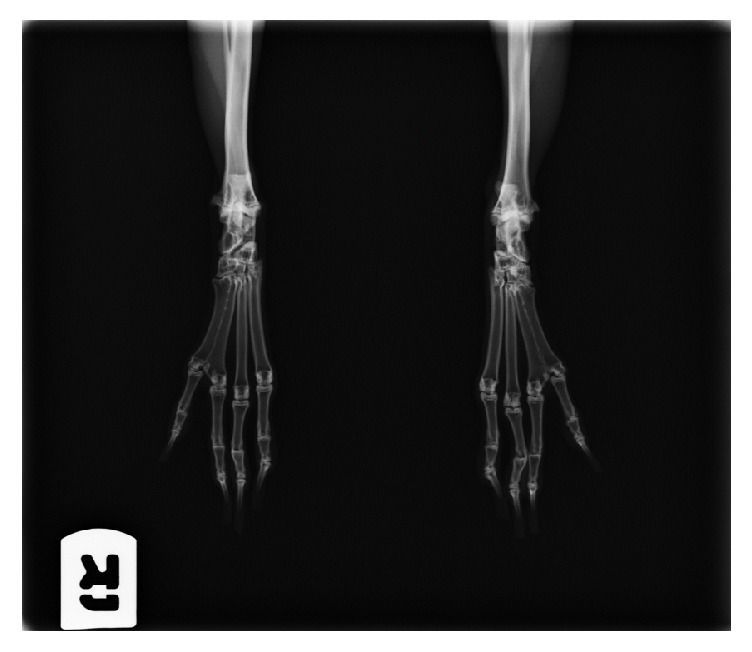
Dorsoplantar view of bilateral syndactyly in a pet rabbit.

The radiological study showed bilateral 3rd and 4th metatarsal bones fused in its entire length preserving normal joint surfaces (Figure 1). The owner was informed of the unusual radiological findings and a comprehensive prechirurgical analytical profile was accepted.

Two blood samples were obtained. The first blood sample was obtained from the right saphenous vein for hematology (MS4 Vet®; Melet Schloesing) and biochemical profile (Chemray 120®; Rayto) and to obtain a serum sample for serology of* Encephalitozoon cuniculi* by indirect immunofluorescence in an external laboratory. The second blood sample was obtained from the left saphenous vein with a special heparinized syringe (PICO 50®; Radiometer) for the evaluation of blood gases and electrolytes in a gasometer (ABL80 Basic® FLEX, Radiometer).

A urinalysis was also performed. The urine was collected with a sterile syringe from a clean surface by manual expression of the bladder. Urinalysis consisted in determination of urine specific gravity by refractometry, protein : creatinine (UP/UCr), and gamma-glutamyl transferase : creatinine (GGTU/CrU) ratios as indicated in the literature [34, 35] and urine strip test (10 Combur test UX®; Roche) with an automatic tester (Urisys®; Roche).

The probes revealed the following alterations (Table 1): hyperglobulinemia, hypercalcemia, and increased UP/UCr. The antibody titer against* Encephalitozoon cuniculi* was positive, 1 : 640 [36].

Orchiectomy and follow-up were uneventful. During anesthesia a complete radiological exam, previously approved by the owner, was performed and no other abnormalities were observed.

## 3. Discussion

The rabbit presented in this case report is the first case of spontaneous syndactyly in a domestic rabbit. Authors have done a comprehensive search in animal dysmorphology databases [37] resulting in the fact that this presented phenotype is not previously reported in rabbits and resembles syndactyly type Ia in humans [37, 38]. Syndactyly was complex, complete, and not complicated and only caused an atypical walking in the rabbit.

Hyperglobulinemia in rabbits has been associated with inflammatory or infectious processes; in this case the encephalitozoonosis can explain this finding [39].

Increased plasma total calcium was not considered relevant because the value of venous ionized calcium (iCa) was within the reference range [40]. Although iCa reference range for domestic rabbits was established in arterial blood it is assumed that there are no significant differences between arterial or venous samples [41, 42].

Raised UP/UCr ratio value (without active sediment) has been associated with renal damage in rabbits [34, 39, 43]. Although positive* Encephalitozoon cuniculi* titer does not always correspond with histological lesions [36] and elevated UP/UCr ratio has not been associated with the parasite in seropositive rabbits by Reusch et al. [34], the parasite can cause kidney damage, whereas it is considered transient and of little relevance [44]. Neither* Encephalitozoon* or other microsporidia have been associated with congenital malformations in animals or people [45].

Due to the absence of symptoms it was decided to evaluate kidney function in later visits, but the owner refused further testing. It is interesting to note that in human medicine syndactyly appears in numerous multiple malformation syndromes, some with renal impairment [10].

The etiology of syndactyly of this rabbit is difficult to assess but is linked to the embryology of the limbs. The development of fingers in rabbits occurs between 14 and 18 days of gestation [28, 46], after the limb bud and the digital rays appear. Although embryology of the limbs is complex and not fully understood, grossly these rays are composed of mesenchymal condensations that will form the digits after separating from each other by cell apoptosis [11, 12]. The authors aim that in that time interval an unknown etiologic agent (e.g., toxic, trauma, or genetic defect) acted. Exposition to environmental toxins or nonprescription products was unlikely in this case. Considering the symmetry of the syndactyly and the suspected etiology in other species a genetic origin is proposed by the authors. Although presented as an isolated malformation, the presence of other malformations in similar cases should be considered by the clinician.

## Figures and Tables

**Table 1:** Relevant laboratory findings in a syndactyly rabbit.

	Value	Reference interval
Hematology		(a)
Biochemistry		
Globulins (g/dL)	3,7^*∗*^	1,5–2,7 (b)
Calcium, total (mg/dL)	14,4^*∗*^	11–14 (b)
Calcium, ionized (mmol/L)	1,83	1,67–1,85 (c)
Urinalysis		
Sediment	Triple phosphate	Triple phosphate, calcium oxalate, calcium carbonate (d)
Proteins (mg/dL)	45,96	7,64–70,37 (e)
Creatinine (mg/dL)	67,45	—
GGT (U/L)	38,25	2,7–96,5 (f)
Ratio PU : CrU	0,68^*∗*^	0,11–0,4 (e)/<0,6 (b)
Ratio GGT : CrU	0,57	0,043–1,034 (f)

^*∗*^Value out of the reference interval.

(a) RBC, WBC, hemoglobin, and hematocrit value were within the reference interval for rabbits by Graham and Mader [47].

(b) Melillo 2007 [39]. ALT, ALP, total protein, albumin, BUN, creatinine, and phosphorus were within the reference interval for rabbits by Melillo [39].

(c) Ardiaca et al. 2013 [42]. Gasometry and electrolyte values (pH, HCO_3−_, BEecf, AnGap, Na, K, and Cl) were within the reference interval for rabbits by Ardiaca et al. [42].

(d) Urine strip values were within the reference interval by Hoefer [43].

(e) Reusch et al. 2009 [34].

(f) Mancinelli et al. 2012 [35].

## Additional Points

The source is Centro Veterinario Madrid Exóticos.
